# Perfluoroalkyl Substances in Plasma of Smallmouth Bass from the Chesapeake Bay Watershed

**DOI:** 10.3390/ijerph18115881

**Published:** 2021-05-30

**Authors:** Vicki S. Blazer, Stephanie E. Gordon, Heather L. Walsh, Cheyenne R. Smith

**Affiliations:** 1 U.S. Geological Survey, Eastern Ecological Science Center-Leetown Research Laboratory, 11649 Leetown Road, Kearneysville, WV 25430, USA; sgordon@usgs.gov (S.E.G.); hwalsh@usgs.gov (H.L.W.); 2 Division of Forestry and Natural Resources, West Virginia University, Morgantown, WV 26506, USA; crsmith@usgs.gov

**Keywords:** perfluoroalkyl compounds, smallmouth bass, plasma, Potomac River, Susquehanna River

## Abstract

Smallmouth bass *Micropterus dolomieu* is an economically important sportfish and within the Chesapeake Bay watershed has experienced a high prevalence of external lesions, infectious disease, mortality events, reproductive endocrine disruption and population declines. To date, no clear or consistent associations with contaminants measured in fish tissue or surface water have been found. Therefore, plasma samples from two sites in the Potomac River and two in the Susquehanna River drainage basins, differing in land-use characteristics, were utilized to determine if perfluoroalkyl substances were present. Four compounds, perfluorooctane sulphonic acid (PFOS), perfluoroundecanoic acid (PFUnA), perfluorodecanoic acid (PFDA) and perfluorododecanoic acid (PFDoA), were detected in every fish. Two additional compounds, perfluorooctane sulphonamide (PFOSA) and perfluorononanoic acid (PFNA), were less commonly detected at lower concentrations, depending on the site. Concentrations of PFOS (up to 574 ng/mL) were the highest detected and varied significantly among sites. No seasonal differences (spring versus fall) in plasma concentrations were observed. Concentrations of PFOS were not significantly different between the sexes. However, PFUnA and PFDoA concentrations were higher in males than females. Both agricultural and developed land-use appeared to be associated with exposure. Further research is needed to determine if these compounds could be affecting the health of smallmouth bass and identify sources.

## 1. Introduction

Per and poly-fluoroalkyl substances (PFAS) have emerged as a global contaminant issue, associated with both human and ecosystem health concerns. These substances have been widely manufactured and used in numerous industrial, agricultural and household products since the 1940s [1,2]. Aqueous film forming foam used at military and civilian airports, train yards, fire training areas, and chemical refineries are common sources of these compounds in the aquatic environment [3]. However, because of their hydrophobic and non-stick properties, they are also widely used in consumer products such as food packaging, cookware, furniture, carpets and outdoor gear [4] and can be present in wastewater treatment plant (WWTP) effluent and sludge/biosolids [5,6,7].

Smallmouth bass *Micropterus dolomieu* is an economically important sportfish in many rivers and streams of North America. While not a major food fish, they may be eaten by anglers. Within the Chesapeake Bay watershed, adult smallmouth bass have experienced disease and mortality events [8] and signs of reproductive endocrine disruption such as testicular oocytes and vitellogenin in males [9,10,11]. Lesions and mortalities of young-of-year smallmouth bass [12,13] are also observed. These disease issues are associated with a variety of pathogens including multiple bacterial, viral and parasitic species [8,13,14,15], suggesting immunomodulation may be occurring.

These observed health issues in smallmouth bass and other fishes led to a multi-site collaborative monitoring program conducted by the U.S. Geological Survey and state agencies in West Virginia, Maryland and Pennsylvania, integrating monthly or biweekly analyses of surface water chemicals with adult smallmouth bass fish health assessments in spring (pre-spawn) and fall (recrudescence) and young-of-year in late spring/early summer from 2013–2019. The data collected are being used to better understand fish health in association with climatic changes, environmental stressors, parasites and pathogens, chemical contaminant exposure and land-use/land management practices. Due to early findings associating testicular oocytes and the total estrogenicity of water samples with agricultural land-use [16,17,18], the chemical monitoring of water focused on pesticides, hormones and phytoestrogens [19]. One contaminant group that has not been addressed in terms of fish health or fish consumption as a source of human exposure within the Chesapeake Bay watershed is PFAS.

Exposure to PFAS has been associated with immune system effects in humans that include reduced antibody concentrations, increased allergies and infections [4,20,21]. Much less information is available for fish immune effects. Experimental studies have shown increased numbers of leukocytes, including granulocytes, lymphocytes and macrophages with increasing concentrations of certain PFAS compounds [22]. Reproductive endocrine disruption, including reduced fecundity, the induction of vitellogenin and testicular oocytes, and differential regulations of reproductive-associated genes have been demonstrated in fishes [23,24,25]. Consequently, we used archived plasma samples to determine (1) if perfluoroalkyl compounds are present in smallmouth bass captured within the Chesapeake Bay watershed; (2) if there are site, sex or seasonal differences in the presence or concentration of plasma compounds; and (3) if there are associations between land-use and plasma concentrations of perfluoroalkyl compounds in this important sportfish.

## 2. Materials and Methods

**Figure 1:**
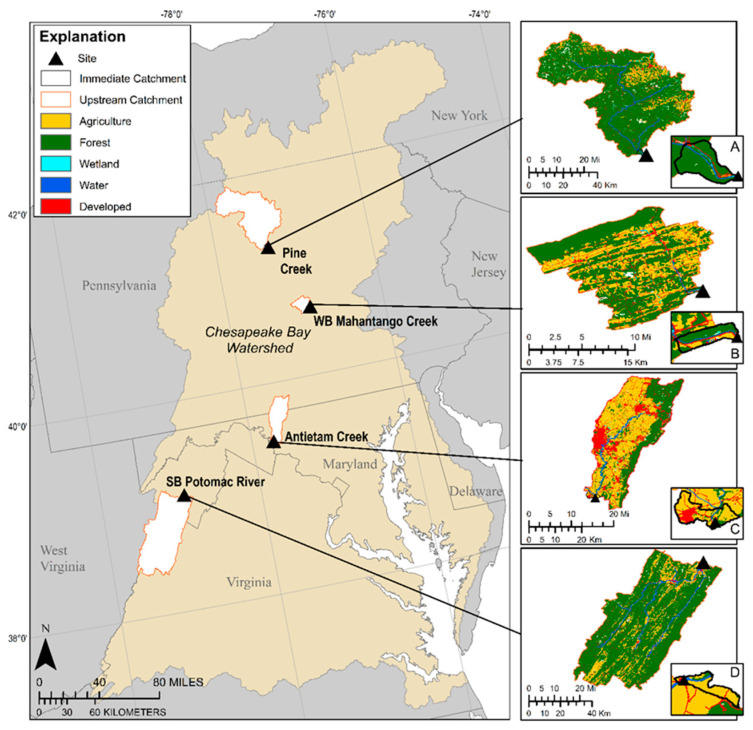
Site locations (Pine Creek, West Branch Mahantango Creek, Antietam Creek, South Branch Potomac River) for smallmouth bass collections and associated land cover in the upstream catchments. Inserts (**A**–**D**) represent the immediate catchments.

Adult smallmouth bass were collected at two sites in the Susquehanna River drainage, Pine and West Branch Mahantango (WB Mahantango) creeks, and two in the Potomac River watershed, Antietam Creek and South Branch Potomac (SB Potomac) River (Figure 1). Pine Creek, the northern-most site, begins in Potter County, Pennsylvania, flowing 140.3 km south to the West Branch Susquehanna River. Fish were sampled within Pine Creek, close to the mouth. WB Mahantango Creek is a 29 km tributary with headwaters in Bald Eagle State Park and joins North Branch Mahantango Creek prior to the confluence with the Susquehanna River. The sampling site was within Mahantango Creek close to the mouth. Antietam Creek is a 67.1 km tributary of the Potomac River, originating in Pennsylvania where the East and West Branches join just north of the Pennsylvania–Maryland border line to form the main stem. It enters the Potomac River south of Sharpsburg, Maryland. Fish were sampled in the Potomac River at the mouth of Antietam Creek. The SB Potomac originates in Highland County, Virginia, flowing through West Virginia, approximately 224 km, to join the North Branch near Green Spring, West Virginia, and form the mainstem Potomac River. The sampling site was near Moorefield, WV, USA.

Land-use characteristics were evaluated at both the immediate and upstream catchments based on the National Hydrography Dataset *Plus* 2 (NHD*Plus*), which integrates the NHD, National Elevation Dataset and the National Watershed Boundary Dataset [26]. The immediate catchment is the stream segment that aligns with the NHD*Plus* framework for the upstream catchment calculations. Landcover data were obtained from the National Land Cover Dataset (NLCD) [27], and 2018 landcover data were summarized using the Zonal Histogram Tool in ArcMap (10.6.1; ESRI, Redlands, CA). The Environmental Protection Agency’s Integrated Compliance Information System (ICIS) National Pollutant Discharge Elimination System (NPDES) data were used to obtain facility locations for all NPDES facilities for each site. Facility locations were displayed in ArcMap (10.6) and spatially joined to the immediate and upstream watersheds. Domestic WWTPs were identified using the keywords “WTP”, “WWTP”, “waste water”, “wastewater”, “water pollution”, “water treatment” “filtration”, “STP”, or “WW Treatment” in the facility name. From the remaining records, the keywords “poultry”, “hatchery”, “CAFO”, “AFO”, “Plant” (except where it coincides with WWTP), “lumber co”, “industrial”, “LLC”, “corp”, “inc.”, “facility”, “mine”, “quarry” or “metal” were used to identify industrial facilities. Remaining facilities were unable to be identified as either domestic or industrial.

Biosolids data were available from the Chesapeake Scenario Assessment Tool (CAST; https://cast.chesapeakebay.net/, (accessed on 12 October 2020) as estimated nutrient (nitrogen and phosphorus) applications from biosolids at the county scale for all counties intersecting the Chesapeake Bay watershed for water year 2018 (1 October–31 September). The combined application rate (pounds/acre) was applied to the acres of turf grass, pasture and cropland per catchment as defined in the 1m High Resolution Chesapeake Landcover (Chesapeake Conservancy; https://www.chesapeakeconservancy.org/conservation-innovation-center/high-resolution-data/land-cover-data-project/, accessed on 12 October 2020). These estimates were summed into total pounds (converted to kg) of nutrients applied for the immediate and upstream catchments to give a final estimate of nutrients from biosolids per catchment.

Bass used in this study were collected by boat electroshocking in the spring (5–14 May) and fall (2–30 October) of 2018. All fish at a particular site were collected on a single day. Fish were held in aerated live wells until processed (less than two hours) and euthanized in 350 mg/L Finquel (MS-222, tricaine methanosulfate, Argent Labs, Redmond, WA) following procedures approved by the U.S. Geological Survey Eastern Ecological Science Center’s Institutional Animal Care and Use Committee. Fish were weighed (gms), measured (total length in mm), examined for visible abnormalities and a blood sample was obtained from the caudal vessels using a sterile 3 mL syringe with a 23-gauge needle. Blood was placed into a heparinized Vacutainer tube (Fisher Scientific, Waltham, MA, USA) and stored on wet ice until returned to the laboratory (2–4 h). Blood was centrifuged at 1000× g at 4 °C for 10 m and plasma was aliquoted into cryovials and stored at −80 C.

Plasma (0.5 mL) samples were shipped on dry ice to SGS AXYS Analytical Services Ltd., Sidney, British Columbia, Canada. We use the term PFAS (poly- and perfluorinated substances) for consistency with the literature; however, 13 perfluoroalkyl analytes (Table 1) were measured by the SGS AXYS Method MLA-042: Analytical Procedure for the Analysis of Perfluoroalkyl Carboxylates and Sulfonates and Perfluorooctane Sulfonamide in Blood Serum by LC-MS/MS. Samples were spiked with isotopically labeled surrogate standards, extracted in formic acid, cleaned up on SPE cartridges and analyzed by liquid chromatography/tandem mass spectrometry (HPLC-MS/MS or UPLC-MS/MS). Final sample concentrations were determined by isotope dilution/internal standard quantification against matrix matched calibration standards carried through the analysis procedure alongside the samples. Results were reported directly in units of ng/mL in the plasma sample. Detection limits were in the 0.5–1 ng/mL range for a 0.5 mL plasma sample.

Statistical analyses were conducted using GraphPad Prism version 9.0.0 (GraphPad Software, San Diego, California). Nonparametric analysis of variance (ANOVA) was completed using the Kruskal–Wallis multiple comparisons test for site comparisons. Unpaired Mann–Whitney test was used to compare sex and seasons within a site. Differences were considered significant at *p* < 0.05.

## 3. Results

### 3.1. Smallmouth Bass Morphometric Characteristics

Plasma samples were available from 34 (17 female, 17 male) smallmouth bass from Antietam Creek, 36 (18 female, 18 male) from SB Potomac, 28 (14 female, 14 male) from WB Mahantango and 32 (15 female, 17 male) from Pine Creek. Fish from Antietam, SB Potomac and Pine Creek were similar in size (total length and weight) and age, while those from WB Mahantango were significantly larger than those collected at the other three sites, and older than those collected at the Antietam and SB Potomac sites (Table 2).

### 3.2. Chemical Detections and Site Comparisons

Perfluorobutanoic acid (PFBA), PFBS, PFHpA, PFHxS, PFHxA, PFOA, and PFPeA were not detected in any samples, while PFDA, PFDoA, PFOS and PFUnA were found in every plasma sample. Perfluorononanoic acid (PFNA) was detected in 29.4% of plasma from Antietam Creek (range BD (below detection) to 1.3 ng/mL), 5.6% from SB Potomac (range BD to 0.7 ng/mL), 3.6% from WB Mahantango Creek (range BD to 0.6 ng/mL) and 12.5% from Pine Creek (range BD to 0.6 ng/mL). Detectable concentrations of PFOSA were observed in 73.5% of plasma samples at Antietam Creek (range BD to 1.0 ng/mL), 0% from SB Potomac, 96.4% at WB Mahantango (range BD to 1.5 ng/mL) and 12.5% at Pine Creek (range BD to 0.5 ng/mL).

Initial site comparisons were made irrespective of sex or season and significant site differences were observed (Table 3). The compound with the highest concentrations at all sites was PFOS. All four sites were significantly different, with Antietam having the highest mean concentrations, followed by WB Mahantango and SB Potomac. The lowest concentrations were observed in fish from Pine Creek. For the other three compounds found in all samples, Antietam also had the highest concentrations. WB Mahantango bass had significantly lower concentrations than Antietam and higher than either SB Potomac or Pine Creek, which were similar. Total plasma perfluoroalkyl compounds followed the same pattern (Table 3).

### 3.3. Seasonal Comparisons

A total of 77 plasma samples from the spring and 53 from the fall were collected. Data from all sites within a season were combined and analyzed. There were no significant differences between the seasons.

### 3.4. Sex Comparisons

All sites and seasons were initially pooled to assess possible sex differences in plasma concentrations of perfluoroalkyl compounds. A total of 64 female and 66 male samples were compared. The mean age (±standard error) of males (4.3 ± 0.2) was not different than females (4.2 ± 0.2). Concentrations of PFDoA (*p* < 0.001) and PFUnA (*p* = 0.005) were higher in males than females, while PFOS and PFDA were not significantly different between the sexes.

**Figure 2:**
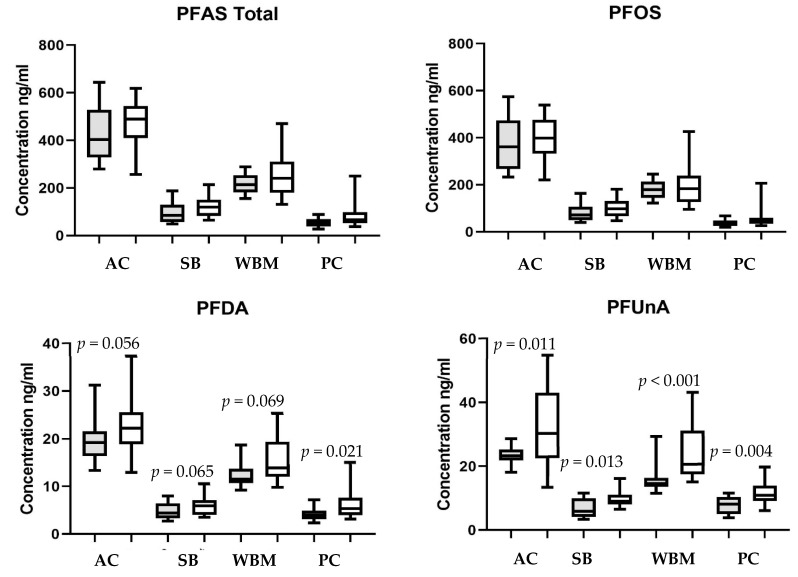

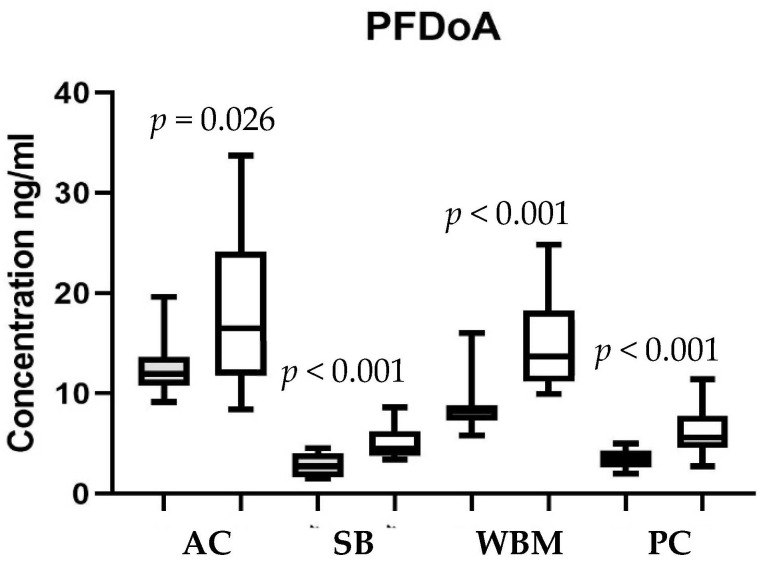
Comparison of perfluoroalkyl substances in female (gray bars) and male (white bars) smallmouth bass plasma from the Antietam Creek (AC), South Branch Potomac (SB), West Branch Mahantango (WBM) and Pine Creek (PC) sites. *p* values indicate difference between male and female at that site. There were no significant differences at any sites for total PFAS or PFOS. Box plots show minimum and maximum values, the median, and interquartile ranges.

Individual site analyses indicated no significant difference between the sexes in PFOS or total PFAS concentrations at any of the sites (Figure 2A,B). Concentrations of PFDA in male bass were only higher (*p* < 0.05) at Pine Creek, while at the other sites the differences were not quite significant (Figure 2C).

Concentrations of PFUnA and PFDoA were significantly higher in males than females at all sites (Figure 2D,E).

### 3.5. Site Land-Use Comparisons

Sites varied in land cover and land-use at both immediate and upstream catchments. In the immediate catchment, the SB Potomac site had the highest percent agricultural land, Antietam Creek was second highest, and WB Mahantango and Pine Creek were lower. Pine Creek was the most forested, followed by WB Mahantango, Antietam Creek and SB Potomac. Antietam Creek was the most developed with 21.4% of the landcover in some level of development, while Pine Creek was the least developed (Table 4).

Contrasting with the immediate catchment, SB Potomac had only 14.0% of the upstream catchment area as agricultural land, with greater forested landcover (Table 5). The upstream catchments at other sites were more consistent with the immediate catchment (Table 5). Pine Creek was again the most forested, and the same pattern of development as noted in the immediate catchment occurred with Antietam Creek being the most developed and Pine Creek and SB Potomac having low levels of development (Table 5).

Nutrient applications from biosolids, which serves as a proxy for biosolids applications, ranged in the immediate catchment from a high of 3.4 kg applied at Pine Creek to zero application at the SB Potomac site (Table 4). However, in the upstream watershed, Antietam Creek had the highest biosolids applications (18,240 kg), while the SB Potomac site still had a relatively small total application of 3.8 kg (Table 5).

Domestic WWTP and industrial discharge facilities were only found in the upstream catchments for all sites. Antietam Creek had the highest number of domestic WWTPs and the second highest number of industrial discharges. SB Potomac had the most industrial discharges and a relatively large number of domestic WWTPs. WB Mahantango had the fewest facility counts, and Pine Creek had the second lowest facilities (Table 5).

## 4. Discussion

To our knowledge, plasma concentrations of PFAS in freshwater sportfish, particularly bass, have not been previously reported in the Potomac and Susquehanna watersheds. Four PFAS compounds, one sulfonic acid (PFOS) and three carboxylic acids (PFDA, PFUnA, PFDoA), were detected in the plasma of every fish, although concentrations varied from site to site and within sites. Two other compounds, PFOSA and PFNA, were detected but at a lower occurrence and concentrations. Previous studies have shown that longer chain compounds tend to bioaccumulate and the sulfonic acid compounds (PFSA) are more bioaccumulative than carboxylic acid compounds (PFCA) with the same carbon chain length [28]. The findings in smallmouth bass were consistent with this as two PFSAs (8 carbons) and four PFCAs (9, 10, 11, 12 carbons) were detected. While these 13 compounds represent a very small proportion of the number of PFAS potentially present in the environment, the findings indicate that additional monitoring needs to occur.

Smallmouth bass from other geographic areas have been reported to contain PFAS [3,29,30,31,32,33]. Unfortunately, these studies utilized fillets (important for establishing human consumption guidelines) or liver and so direct comparisons cannot be made. A study in New York state waterbodies found that liver PFOS concentrations ranged from 10 to 431 ng/g wet weight, with higher concentrations in males than females. In the current study, there were no sex differences in PFOS plasma concentrations; however, males did have higher concentrations of PFUnA and PFDoA. Conversely, in laboratory exposures, female fathead minnow accumulated higher levels of PFOS than males [34], illustrating effects of species, tissues, behavior and exposure route. For instance, longer chained PFAS have been shown to accumulate in sediments [35]. In the spring, it is the male bass that have increased exposure to sediments as they build and guard the nests.

The relationship between PFAS concentrations in blood/plasma and liver or muscle in smallmouth bass is currently unknown. Plasma/blood provides a potential non-lethal sample, may provide better insights into fish health effects and can be sampled repeatedly from the same individual [36]. It also provides a comparison to human exposure/toxicity as most human studies utilize blood/plasma/serum. Laboratory and field studies suggest blood or plasma has the highest concentrations when compared to other tissues, in many but not all fishes. The distribution of PFOS in tiger puffer fish (*Takifugu rubripes*) after a single intraperitoneal injection of 0.1 mg/kg body weight documented plasma levels over 700 ng/mL on day 1 and 861 ng/mL at 14 days. Mucus, suggested as an elimination route, had the next highest (690 ng/g) and gradually increased over time. Liver had the third highest concentrations [37]. Tissue distribution and accumulation were examined in rainbow trout (*Oncorhynchus mykiss*) exposed through the water. The greatest PFOS concentrations were in blood and 94–99% were in plasma versus the cellular fraction. The lowest concentrations were in muscle [38]. In aqueous lab exposures of fathead minnow, PFOS concentrations were highest in the blood, followed by liver and gonad [34]. However, in a study of multiple wild fish species from various sites in Japan, ratios of PFOS between liver and blood varied widely (0.02 to 179) among species [39]. In the future, it will be important to determine the tissue distribution of PFAS in smallmouth bass.

Laboratory studies suggest PFOS accumulation in fishes is primarily through aqueous exposure and dietary sources are secondary [34]. However, the assessment of bioaccumulation factors (combined effects of all uptake pathways) in wild fishes does not necessarily support that finding. Many factors, including PFOS precursors and temporal variability, can influence field-based bioconcentration factors (the direct uptake of a chemical by an organism from water or air), which are generally an order of magnitude higher than controlled laboratory exposures [28,40]. In one study, a bioconcentration factor of 8850 was estimated between smallmouth bass liver and surface water [33]. A survey of freshwater fishes from urban rivers and Great Lakes sites indicated that pooled fillet samples from carnivorous species tended to be higher than other species [31]. Fish species collected from a lake receiving discharge from a WWTP in Beijing, China, varied in concentrations, somewhat related to trophic level [41]. Smallmouth bass feed on aquatic insects and zooplankton as young, shifting to predominately fish, crayfish and other prey, and are considered top predators as adults [42]. Hence, both water and diet may play a role in PFAS concentrations in smallmouth bass.

Concentrations of plasma PFAS, particularly at the Antietam Creek site, were high compared to other wild fish species for which blood/plasma data are available in the literature (Table 6). One exception was in bluegill *Lepomis macrochirus* from Lake Biwa, Japan’s largest lake with a high degree of surrounding development. Blood concentrations of 455–834 ng/mL were measured in bluegill, while largemouth bass *M. salmoides* from the same lake had lower concentrations (317–322 ng/mL). Unfortunately, only two fish of each species were analyzed and no information on fish size or age was provided [39]. Striped bass *Morone saxatilis* collected from the Cape Fear River, North Carolina, downstream of a PFAS production facility had plasma concentrations of PFOS up to 977 ng/mL [43].

**Figure 3:**
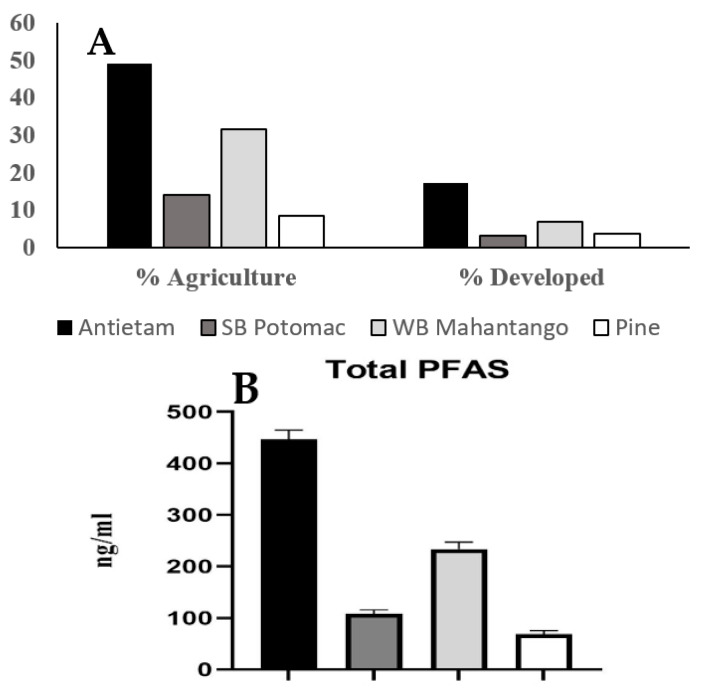
(**A**) Pattern among the sites in % agriculture and % developed land-use in the upstream catchment. (**B**) Pattern of total PFAS compounds measured in smallmouth bass plasma.

The four sites in this study were part of a long-term monitoring project primarily initiated to assess the effects of emerging contaminants from agricultural practices on the general and reproductive health of smallmouth bass. Hence, these subwatersheds had varying types and intensity of agricultural land-use and a relatively low (<25%) level of urbanization (developed). There are likely multiple sources of PFAS in these watersheds. The pattern among sites for total PFAS and the four compounds measured in all fish was the same as the pattern of both agricultural and developed land-use in the upstream catchment (Figure 3). The Antietam Creek site had the highest percentage of both agricultural and developed land-use, as well as the highest number of domestic WWTPs. In the nearby Delaware River, higher concentrations were generally measured in the more developed zones. The same six analytes we detected were also detected in fish fillets from the Delaware River, with nontidal smallmouth bass having the highest PFOS concentrations of the four species tested [29].

Domestic WWTPs, industrial wastewater and biosolid application may all be sources of PFAS in this study. A better understanding of scale (immediate versus upstream), transport, types of point sources and types of treatments within individual WWTPs in a catchment will be necessary to better manage exposure. For instance, a comparison of samples taken at various stages of treatment in a WWTP demonstrated that PFOS was 3-fold higher in the outflow than the inflow and 8-fold higher in waste-activated sludge than in primary sludge [5]. The use of these biosolids in agriculture can provide a source of PFAS through run-off to surface water or by infiltration to groundwater [7,46]. The type of wastewater facility is also important. In one study, the highest release of PFAS was from WWTPs with industrial wastewater, second highest in small domestic WWTPs also receiving commercial and industrial wastewater and lowest in plants with only domestic wastewater [47]. PFOS, PFDA, PFUnA and PFNA were detected in effluents from both domestic and industrial source types, although higher in industrial effluents. The highest emissions were from the metal and paper industry. Concentrations of PFDoA were higher in domestic WWTP effluent and PFOSA was only detected in WWTP effluent [47].

## 5. Conclusions

The results of this study indicate that PFAS are accumulating in smallmouth bass, an important sportfish, captured in the Potomac and Susquehanna rivers, located within the Chesapeake Bay watershed. Four compounds were detected in every smallmouth bass plasma sample and were particularly high at the Antietam Creek site, the site with the highest developed and agricultural landcover in the upstream catchment. Total plasma PFAS ranged from 256 to 644 ng/mL at this site and were higher than plasma or blood concentrations at most sites and species previously reported in the literature. The four sites varied significantly in concentrations, which was likely due to land-use within the catchments. It is currently not known if these levels may be associated with impaired health and reproduction observed in smallmouth in this region. The results suggest further research is needed to identify sources and to determine if biological effects are associated with environmentally relevant exposure to these compounds. The latter is particularly important since little is known about the effects of PFAS exposure and tissue accumulation in wild fish species.

## Figures and Tables

**Table 1:** Perfluoroalkyl substances analyzed in smallmouth bass plasma.

Chemical Name	Abbreviation	Carbon Chain Length
*Perfluoroalkyl Carboxylic Acid Compounds*		
Perfluorobutanoic acid	PFBA	4
Perfluoro-n-pentanoic acid	PFPeA	5
Perfluorohexanoic acid	PFHxA	6
Perfluoroheptanoic acid	PFHpA	7
Perfluorooctanoic acid	PFOA	8
Perfluorononanoic acid	PFNA	9
Perfluorodecanoic acid	PFDA	10
Perfluoroundecanoic acid	PFUnA	11
Perfluorododecanoic acid	PFDoA	12
*Perfluoroalkyl Sulfonic Acid Compounds*		
Perfluorobutane sulfonic acid	PFBS	4
Perfluorohexane sulfonic acid	PFHxS	6
Perfluorooctane sulfonamide	PFOSA	8
Perfluorooctane sulfonic acid	PFOS	8

**Table 2:** Morphometric characteristics of smallmouth bass collected at four sites within the Chesapeake Bay watershed.

Site	Sample Size	Length (mm) ^1^	Weight (gm) ^1^	Age (Years) ^1^
Antietam Creek	34	304 ± 40 ^a^	375 ± 169 ^a^	3.9 ± 1.2 ^a^
South Branch Potomac	36	309 ± 57 ^a^	436 ± 258 ^a^	4.0 ± 1.5 ^a^
West Branch Mahantango	28	368 ± 37 ^b^	694 ± 259 ^b^	5.0 ± 1.8 ^b^
Pine Creek	32	280 ± 43 ^a^	314 ± 135 ^a^	4.2 ± 1.0 ^a,b^

^1^ Presented as mean ± standard error. (^a,b^) Values followed by the same letter are not significantly different.

**Table 3:** Site comparisons of the perfluoroalkyl chemical concentrations detected in smallmouth bass. Presented as mean ± standard error with minimum and maximum below in parentheses.

Site	Sample Size	PFOS ng/mL	PFUnA ng/mL	PFDA ng/mL	PFDoA ng/mL	Total ^1^ PFAS ng/mL
Antietam Creek	34	381.4 ± 16.5 ^a^ (220–574)	28.4 ± 1.7 ^a^ (13–55)	21.4 ± 1.0 ^a^ (13–37)	15.1 ± 1.1 ^a^ (8–34)	447.0 ± 17.8 ^a^ (256–644)
South Branch Potomac	36	90.4 ± 6.5 ^c^ (40–181)	8.4 ± 0.5 ^c^ (3–14)	5.3 ± 0.3 ^c^ (3–11)	4.0 ± 0.3 ^c^ (1–9)	108.2 ± 7.5 ^c^ (49–214)
West Branch Mahantango Creek	28	187.1 ± 12.6 ^b^ (95–427)	20.2 ± 1.5 ^b^ (12–43)	14.1 ± 0.8 ^b^ (9–25)	11.8 ± 0.9 ^b^ (6–25)	234.0 ± 3.3 ^b^ (131–470)
Pine Creek	32	48.4 ± 5.8 ^d^ (20–206)	9.8 ± 0.6 ^c^ (4–20)	5.2 ± 0.4 ^c^ (2–15)	4.9 ± 0.5 ^c^ (2–11)	68.6 ± 7.0 ^c^ (28–250)

^1^ Includes PFNA and PFOSA when detected; ^a,b,c^ Values within a column followed by the same letter are not significantly different.

**Table 4:** Land-use characteristics of the smallmouth bass sampling sites at the immediate catchment scale.

Characteristics	Antietam Creek	South Branch Potomac River	West Branch Mahantango Creek	Pine Creek
Drainage Area (km^2^)	5.95	1.04	1.21	8.49
Percent Agriculture	63.6	74.4	20.4	1.1
Percent Forest	14.1	10.4	66.1	88.2
Percent Developed	21.4	7.2	10.0	4.2
Nutrients from Biosolids (kg)	0.1	0	2.9	3.4
Domestic Wastewater Treatment Plants	0	0	0	0
Industrial Wastewater Facilities	0	0	0	0

**Table 5:** Land-use characteristics of smallmouth bass sampling sites in the upstream cumulative catchment.

Characteristics	Antietam Creek	South Branch Potomac River	West Branch Mahantango Creek	Pine Creek
Drainage Area (km^2^)	730	3151	218	2437
Percent Agriculture	49.1	14.0	31.6	8.5
Percent Forest	32.2	80.6	60.1	84.2
Percent Developed	17.3	3.2	7.0	3.6
Nutrients from Biosolids (kg)	18,240	3.8	799	1486
Domestic Wastewater Treatment Plants	27	13	1	7
Industrial Wastewater Facilities	51	77	9	12

**Table 6:** Comparison of plasma, serum or blood perfluoroalkyl concentrations in fish species worldwide as reported in the literature.

Species	Range (ng/mL)		Citation
Smallmouth bass *Micropterus dolomieu*	PFOS	20–574	Current study
	PFDA	2–37	
	PFUnDA	3–55	
	PFNA	BD–1.3	
Chub *Leuciscus cephalus*	PFOS	38.9–57.8	[44]
	PFNA	0.88–7.1	
Grey mullet *Mugil cephalus*	PFOS	93 (mean)	[45]
	PFDA	13 (mean)	
	PFNA	3.8 (mean)	
	PFUnDA	26 (mean)	
Rockfish *Sebastes inermis*	PFOS	31 (mean)	[45]
	PFDA	1.9 (mean)	
	PFNA	1.9 (mean)	
	PFUnDA	3.6 (mean)	
Crucian carp *Carassius auratus*	PFOS	48.9–84.4	[41]
	PFDA	9.7–25.4	
	PFUnDA	7.0–15.3	
	PFOSA	0.3–2.0	
	PFNA	0.3–1.2	
Common carp *Cyprinus carpio*	PFOS	14.2–32.2	[41]
	PFDA	1.4–11.7	
	PFUnDA	1.1–9.0	
	PFNA	0.1–0.7	
White semiknife carp *Hemiculter leucisculus*	PFOS	8.4–11.4	[41]
	PFDA	5.2–10.3	
	PFUnDA	1.7–2.5	
	PFOSA	1.0–5.8	
	PFNA	0.3–0.5	
Nile tilapia *Oreochromis niloticus*	PFOS	4.8–6.7	[41]
	PFDA	2.9–4.3	
	PFUnDA	1.6–1.9	
	PFOSA	0.1–2.8	
	PFNA	0.3–0.5	
Leather catfish *Clarias lazera*	PFOS	7.0–25.9	[41]
	PFDA	5.1–15.5	
	PFUnDA	5.4–14.1	
	PFNA	0.1–1.0	
Largemouth bass *Micropterus salmoides*	PFOS	317–322	[39]
Blue gill *Lepomis macrochirus*	PFOS	455–834	[39]
Common carp *Cyprinus carpio*	PFOS	68–77	[39]
Striped bass *Morone saxatilis*	PFOS	4.6–977	[43]
	PFDA	1.7–146	
	PFNA	0.3–11.6	

## Data Availability

Data will be available in a ScienceBase data release at https://doi.org/10.5066/P9H8DW78. accessed on 10 June 2021.
